# Research progress of postoperative cognitive dysfunction in cardiac surgery under cardiopulmonary bypass

**DOI:** 10.1002/ibra.12123

**Published:** 2023-08-14

**Authors:** Yi‐Ming Zhuang, Ji‐Yang Xu, Kun Zheng, Hong Zhang

**Affiliations:** ^1^ Department of Anesthesiology Affiliated Hospital of Zunyi Medical University Zunyi China; ^2^ Department of Anesthesiology Judicial Police Hospital of Guizhou Province Guiyang China; ^3^ Department of Anesthesiology Guizhou Provincial People's Hospital Guiyang China

**Keywords:** cardiac surgery, cardiopulmonary bypass, perioperative neurocognitive disorder, postoperative cognitive dysfunction

## Abstract

Cardiopulmonary bypass (CPB) is often used in cardiothoracic surgery because its nonphysiological state causes pathophysiological changes in the body, causing multiorgan and multitissue damage to varying degrees. Postoperative cognitive dysfunction (POCD) is a common central nervous system complication after cardiac surgery. The etiology and mechanism of POCD are not clear. Neuroinflammation, brain mitochondrial dysfunction, cerebral embolism, ischemia, hypoxia, and other factors are related to the pathogenesis of POCD. There is a close relationship between CPB and POCD, as CPB can cause inflammation, hypoxia and reperfusion injury, and microemboli formation, all of which can trigger POCD. POCD increases medical costs, seriously affects patients' quality of life, and increases mortality. Currently, there is a lack of effective treatment methods for POCD. Commonly used methods include preoperative health management, reducing inflammation response during surgery, preventing microemboli formation, and implementing individualized rehabilitation programs after surgery. Strengthening preventive measures can minimize the occurrence of POCD and its adverse effects.

## Graphical abstract

Cardiopulmonary bypass (CPB) is used in cardiothoracic surgery and leads to pathophysiological changes in the body, causing multiorgan and multitissue damage and frequently leading to central nervous system complications. Here, we review the latest advances in CPB and postoperative cognitive dysfunction (POCD) in the last 3 years and analyze the pathogenesis, risk factors, detection indexes, and other factors to provide better ideas for healthcare workers to better understand the development of POCD and reduce postoperative cognitive dysfunction in patients undergoing cardiac surgery, ultimately enabling to benefit patients.

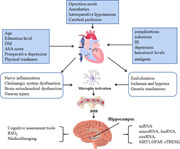

## 1 INTRODUCTION

Early and long‐term postoperative neurological complications caused by central nervous system injury are still the most dangerous factors affecting the survival and prognosis of patients undergoing cardiac surgery.^1^ Cardiothoracic surgery is a common surgical method and a life‐saving procedure, but various complications usually accompany it. Apart from surgical trauma, Cardiopulmonary bypass (CPB) technology is a process of draining blood from the heart, which can lead to pathological and physiological changes in multiple organs and tissues. In the decades after the birth of CPB, the complications of CPB have gradually decreased through continuous technological updates.^2^ However, 33%–83% of CPB patients have Postoperative cognitive dysfunction (POCD) after cardiac surgery.^3^

Perioperative neurocognitive disorder (PND) includes preoperative neurocognitive impairment, POCD, and postoperative delirium (POD). POCD refers to a decline in cognitive function, such as memory, attention, and executive function, following surgery. PND has recently been recommended to define perioperative cognitive decompensation, a common neurological complication after surgery and anesthesia, and is particularly common in elderly patients postoperatively, increasing the risk of adverse outcomes in the postoperative period.^4^ With the increasing aging of the population and advances in medical and surgical modalities, the number of elderly surgical patients is increasing.^5^ The number of occurring PNDs has also increased, and patients with PNDs have longer hospital stays and greater healthcare expenditures, posing significant challenges to society and the healthcare system.^6^ Although there are many clinical differences between POD and POCD, they are both mediated by a systemic inflammatory response triggered by surgical trauma, which leads to blood–brain barrier (BBB) dysfunction and promotes neuroinflammation.^7^ The incidence of cognitive decline in patients undergoing coronary artery bypass grafting (CABG) using the CPB technique was 53% at hospital discharge and remained as high as 42%, 5 years after surgery.^8^ Other studies have also suggested that cardiac surgery has a more severe impact on cognitive performance than other sites, such as abdominal surgery.^9^

**Figure 1:**
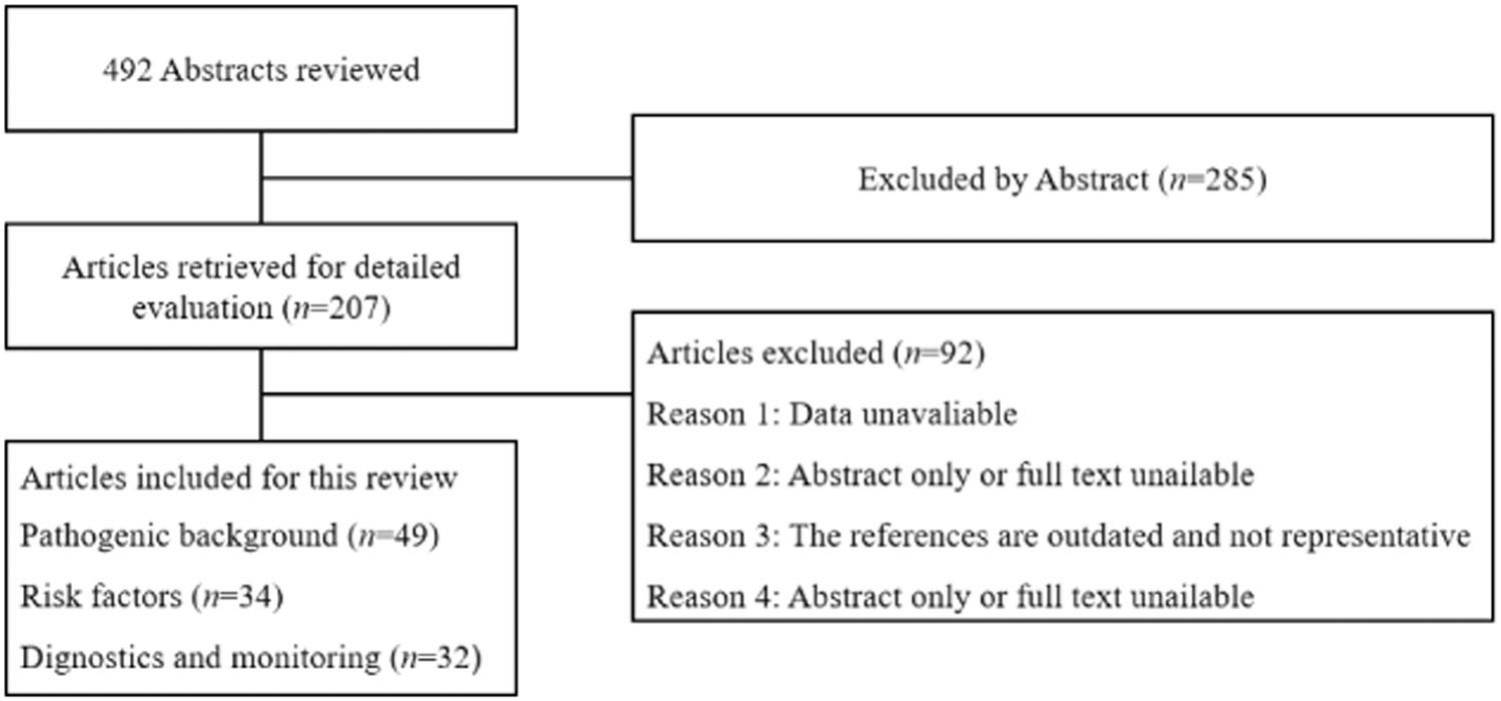
The identification and selection of studies included in this narrative review.

This review systematically searched the literature of Web of Science, PubMed, and Embase to February 2023 and analyzed the pathogenesis, risk factors, detection indicators, and research progress of POCD using the subject words “PND, POCD, CPB, and cardiac surgery” in MeSH. We reviewed 492 abstracts, retrieved 207 articles, and ultimately selected 120 (Table 1, Figure 1). It provides a better idea for medical workers to understand the occurrence and development of POCD and reduce POCD in cardiac surgery patients.

**Table 1:** Literature search and selection.

Keywords	Number of documents retrieved		
	All	Finally included	Included in recent 3 years
Perioperative neurocognitive disorder, postoperative cognitive dysfunction, cardiopulmonary bypass, cardiac surgery	492	114	90

## 2 THE PATHOGENESIS OF POCD

The apoptosis of hippocampal cells caused by CPB is the main feature that impairs cognitive function. Advanced age results in decreased physical function, anesthesia interference, surgical stimulation, and other factors that can cause oxidative stress (OS), vascular embolism, decreased permeability, ischemia, and hypoxia. They can also result in dysfunction of the cholinergic system, a decrease in nerve growth factor (NGF), impaired mitochondrial function, neuronal degeneration, inflammatory neuropathy, neuronal apoptosis, reduced numbers of neurons, all of which are related to the onset and increased risk of POCD (Table 2).^10^, ^11^, ^12^

**Table 2:** The pathogenesis of postoperative cognitive dysfunction (POCD).

Type of pathogenesis	Characteristics
Nerve inflammation	Surgery‐induced hippocampal inflammation is the main cause of POCD^13^
Cholinergic system dysfunction	The central cholinergic anti‐inflammatory pathway is related to consciousness, learning, and memory formation^14^
Brain mitochondrial dysfunction	Neuronal function and neuronal survival are directly related to mitochondria^15^
Neuron injury	Reducing neuronal injury can reduce the incidence of postoperative delirium^16^
Embolization	Important causes of POCD^17^
Ischemia and hypoxia	Cerebral perfusion and oxygen supply are the most important predictors^18^
Genetic mechanism	Changes in gene level play a key role in the pathogenesis of POCD^19^

### 2.1 Cholinergic system dysfunction

The central cholinergic anti‐inflammatory pathway (CAP) plays an important role in the pathogenesis of POCD. The central cholinergic nervous system is related to consciousness, learning, and memory formation. It can synthesize and secrete the essential protein for neuron growth and survival: neurotrophic factor (NGF), which supports the integrity of neuronal growth, development, and function. In the mouse model of cognitive impairment induced by sevoflurane, the expression of silence‐specific protein 1 (SP1) can save the inactivation of CAP in the hippocampus of rats, reduce neuroinflammation and apoptosis, and reduce the development of POCD.^14^ After the operation, the number of cholinergic neurons in the basal forebrain decreased significantly, the activity of acetylcholine transferase in the hippocampus and amygdala decreased, and the acetylcholine content decreased.^20^ Clinical use of cholinesterase inhibitor physostigmine has improved postoperative cognitive function.^21^ Hippocampal RNA‐seq of mice inhaled sevoflurane also found that clearly, expressed signal pathways included cholinergic pathways.^22^ The importance of CAP in addressing neuroinflammation and cognitive impairment caused by aseptic trauma has been recognized. The excessive and persistent decline in cognitive function and inflammatory response in elderly mice are associated with CAP dysfunction.^23^

### 2.2 Brain mitochondrial dysfunction

Many studies have shown that neuronal function and survival are directly related to mitochondria. The pathophysiological changes of memory impairment include abnormal mitochondrial function, OS, inflammatory response, nerve cell apoptosis, and loss of hippocampal dendritic spines. Mitochondrial dysfunction is an important feature of degenerated neurons.^15^ When dynamin‐related protein 1 (DRP1), an important regulator of mitochondrial dynamics, is activated in primary neurons, mitochondrial breakage increases.^24^ During deep hypothermic cardiac arrest (dHCA), proteins protect nerves from injury through local changes, inducing differential expression of mitochondrial proteins in the hippocampus of 29 kinds of rats.^25^ Hippocampal OS disorder induced by sevoflurane can regulate mitochondrial function and autophagy through SP1. Inhibition of mitochondrial permeability transition (MPT) pore and subsequent apoptosis pathway has a neuroprotective effect. Sirtuin‐3 (SIRT3), a NAD‐dependent deacetylase protein located in mitochondria, is the only sirtuin protein found to specifically play a role in prolonging the human lifespan. It also prevents cognitive decline in aged mice induced by anesthesia or surgery by regulating mitochondrial function and inhibiting hippocampal nerve inflammation.

### 2.3 Nerve inflammation

Many clinical and preclinical studies have confirmed a correlation between neuroinflammation and POCD.^26^ The main effect of surgery on POCD is that it induces hippocampal inflammation.^13^

Long‐term anesthesia can activate NF‐κB inflammatory pathway, cause nerve inflammation, inhibit neuronal excitability, and appear cognitive dysfunction and anxiety‐like behavior.^27^ The biomarkers of inflammation in cerebrospinal fluid (CSF) and blood increased significantly after the operation.^28^ The expression of plasma inflammatory cytokines NOD‐, LRR‐, and pyrin domain‐containing protein 3 (NLRP3) in the hippocampus of mice increased after a cardiac operation, which caused hippocampal inflammation and cognitive dysfunction.^29^, ^30^ The key site of inflammation‐mediated nerve injury is still unclear,^24^ but there is evidence that the locus coeruleus noradrenergic system (LC‐NE) system plays a regulatory role in neuroinflammation in POCD rats.^31^ In the prefrontal cortex of aged rats, it has been proved that activation of autophagy can inhibit the formation of NLRP3 inflammatory bodies and alleviate the cognitive impairment of aged rats. POD and POCD were also closely related to the concentration of peripheral inflammatory markers. The significant increase of inflammatory biomarkers C‐reactive protein (CRP) and interleukin‐6 (IL‐6) was positively correlated with the type of operation.^32^ In the experiment, the neurons of rats inhaled anesthetics showed obvious inflammation and apoptosis.^14^, ^33^ The dysfunction of the BBB further aggravates the occurrence of neuroinflammation. A large number of differentially expressed genes were found by RNA‐Seq detection in the inflammatory hippocampus.^34^ Microglia mediate hippocampal inflammation and cognitive decline in mice after the operation.^35^ Natural killer cells can show severe inflammation in nervous system diseases and inhibit overactivated microglia.^36^ It is believed that the intensity of CPB cardiac surgery in elderly patients does not affect the development of early postoperative POCD.^37^ However, for the safety of patients and long‐term prognosis, minimize surgical trauma to reduce the postoperative inflammatory reaction and the incidence of POCD as much as possible.^38^

### 2.4 Neuron injury

Central inflammatory reaction, neuron apoptosis, and a decrease in the total number of neurons play an important role in the occurrence and development of POCD. In 400 patients undergoing cardiac surgery, it was found that POD was associated with increased neuronal damage, especially when the BBB was destroyed.^39^ The Rat CPB‐POCD model also proved that CPB impaired cognitive function, induced hippocampal cell apoptosis, and inhibited hippocampal cell apoptosis. Reducing excitotoxic injury, regulating apoptotic proteins, and interfering with inflammatory response can reduce nerve cell injury after ischemia and the occurrence of POD.^16^ Hypoxia/reoxygenation inhibited the vitality of hippocampal neurons and increased cell apoptosis.^40^ Autophagy was significantly inhibited after sevoflurane exposure, and the activation of NLRP3 inflammatory bodies caused more severe nerve cell damage.^41^

### 2.5 Ischemia and hypoxia

Cerebral perfusion and oxygen supply are the most important predictors of postoperative brain injury, whether in the perioperative or during CPB. Low cerebral perfusion, hypoxia, and inflammatory reaction can lead to brain injury. Brain function is easily affected by hypoxemia. When ischemia‐reperfusion injury occurs, the change in BBB permeability leads to increased vascular‐derived substances in brain tissue, aggravating neuronal damage.^18^ Studies have shown cerebral oxygenation damage during cardiothoracic surgery in elderly patients is related to their poor neurological prognosis, and cerebral hypoxia is a common neurological complication after cardiac surgery. In animal experiments, cognitive impairment occurred significantly in rats who received a 2‐h CPB operation. Cerebral thrombosis and hypoxia occurred in the hippocampal CA3 region of rats after an operation. Microglia in the hippocampus is activated, the expression of inflammatory factors such as IL‐1β, IL‐6, and TNF‐α is upregulated, and the permeability of the BBB is increased.^42^ Although it is controversial to determine whether the intervention guided by intraoperative monitoring of cerebral oxygen saturation can reduce the risk of POCD, many studies have shown that the decrease of intraoperative cerebral oxygen saturation is related to the increased risk of POD. Comprehensive assessment of regional cerebral oxygen saturation (rSO_2_) and guidance for intervention in the risk of POD and POCD can effectively shorten the length of stay in the intensive care unit (ICU) in adults undergoing cardiac surgery.^43^

### 2.6 Embolization

Left atrial thrombus is an independent risk factor for PND after cardiac surgery,^44^ and cerebral microemboli during CPB is also one of the important causes of POCD. In a study of CPB and off‐pump surgery in 168 patients, middle cerebral artery microemboli were continuously measured by bilateral transcranial Doppler ultrasound. It was found that avoiding CPB in CABG significantly reduced the number of cerebral microemboli, but the incidence of POCD did not decrease 1 week or 3 months after CABG.^17^ Katarzyna analyzed seven articles describing neuropsychological studies in 2796 patients and concluded that there was no significant correlation between CPB or brain microemboli and the occurrence of POCD.^45^ However, we believe that ignoring POCD and microemboli exposure would be unwise, as microemboli have been demonstrated to be harmful in both experimental and clinical settings, and minimizing patient exposure to them remains of paramount importance.

### 2.7 Genetic mechanism

Through the study of 16‐month‐old humanized apolipoprotein E targeted replacement mice carrying apolipoprotein E gene 3 (apoE3) or APOE4, it was found that APOE4 aggravated systemic inflammatory response, APOE4 was the susceptible gene of POCD, the neurocognitive and behavioral effects of operation and anesthesia depended on the baseline neurocognitive state, and the changes of gene level caused by operation and anesthesia played a key role in the pathogenesis of POCD.^19^

## 3 PREOPERATIVE RISK FACTORS FOR POCD

Cognitive dysfunction assessed preoperatively was associated with poor postoperative outcomes. c‐POCD was well predicted by Xie et al. by recruiting 265 patients undergoing cardiac surgery by analyzing the risk of preoperative and intraoperative CPB time, hypertension, white blood cell count, aspartate amino transferase (AST), and arrhythmias.^46^ Preoperative risk factors were also associated with increasing age, body mass index (BMI), male, ethnicity, decreased functional independence and decreased metabolic functional capacity, and patients with a history of stroke or transient ischemic attack, chronic obstructive pulmonary disease, diabetes, cirrhosis, and alcohol abuse were more likely to develop cognitive dysfunction.^47^

### 3.1 Age

Age is an independent risk factor for PND after cardiac surgery.^17^, ^44^ With the increase in age, the proportion of postoperative POCD is higher.^5^, ^48^ Age has become the most common predictor of cognitive outcome.^49^, ^50^ It is necessary to strengthen the preoperative screening, intraoperative management, and postoperative follow‐up of elderly patients. Acute changes in mental state are characterized by impaired consciousness, decreased attention, and cognitive changes, and delirium is very common in elderly and hospitalized patients, especially after major surgery, which increases the morbidity and mortality of patients.^51^ With the progress of technology, the mortality rate associated with CABG surgery is decreasing, but the proportion of PND and POCD is still high,^52^ which is an important risk factor for the long‐term deterioration of coronary artery disease.^53^

### 3.2 Education level

Low education is a risk factor for early POCD.^50^ A study found that the degree of weakness with a low education level ≥60 years old is significant.^54^ Tracey and other adult patients who have undergone open heart surgery in the past 6 years have indicated that elderly patients and patients with low education levels are prone to POCD.^49^

### 3.3 Diabetes mellitus (DM)

Diabetes is an independent risk factor for PND, and patients with diabetes are prone to abnormal cognitive screening.^47^ Experiments in rat PND models have also confirmed elevated Aβ and p‐tau and significantly higher apoptosis rates in hippocampal cells in rats treated with diabetes or surgery.^55^

### 3.4 American Society of Anesthesiology (ASA) score

Patient physical status ASA classification score III–V has emerged as an independent risk factor for recovery room delirium (RRD), resulting in longer preoperative and postoperative hospital stays and higher medical costs.^56^ A prolonged preoperative hospital stay may lead to increased POCD.^57^ Patients with higher ASA classification scores have more debilitating physical status tests and a higher chance of delirium.^58^

### 3.5 Others

Preoperative depression is a risk factor for acute cognitive decline and a moderate risk factor for cognitive decline 1–6 months after CABG, becoming one of the important risk factors for POCD after CABG.^52^ Physical weakness is also a risk factor for complications of POD and POCD,^58^ and the occurrence of POCD will further worsen the underlying patient condition.

## 4 INTRAOPERATIVE RISK FACTORS FOR POCD

Type of surgery, duration, and method of anesthesia. Duration of CPB and ascending aortic block (AAB), hypertension, leukocyte count, AST, and arrhythmias were associated with cognitive dysfunction after cardiac surgery.^46^

### 4.1 Operation mode

POCD is common and severe after cardiac surgery, so special attention should be paid to the choice of surgical approach. Open procedures such as mitral valve surgery and aortic valve surgery occur more POCD than CABG, and perivalvular fistula (PVL) often occurs after aortic valve replacement (TAVI), which negatively affects both short‐ and long‐term survival.^59^ After CPB cardiac surgery, a significant proportion of patients experience transient visual hallucinations.^60^ Current studies have shown that minimally invasive surgery to lower blood pressure can reduce the incidence and severity of POCD, tissue trauma, and postoperative inflammatory response.^38^ Although CPB and cerebral microemboli have been reported to be unrelated to the risk of POCD, it is still important to minimize the occurrence of microemboli during surgery to improve the safety of CPB.^29^, ^61^

### 4.2 Anesthetic factors

Postoperative cognitive function is deeply affected by anesthesia, and prolonged anesthesia‐induced neurotoxicity is prone to PND, especially in elderly surgical patients. Sevoflurane‐induced neurotoxicity (SIN) triggers the activation of the NF‐κB inflammatory pathway, and neuronal excitability is inhibited. Cognitive dysfunction and anxiety‐like behavior appear,^16^ causing acute effects and blocking long‐term potentiation (LTP), a cell associated with learning and memory. Conversely, neuroprotective activities protect patients from poor memory and possible neurodegeneration.^62^ Sevoflurane exposure affected the expression of inflammation index and autophagy protein in the prefrontal cortex of elderly rats, NLRP3 protein expression was significantly upregulated, inflammatory cytokines IL‐1β and IL‐18 expression and mRNA expression increased,^41^ and SP1 could regulate sevoflurane‐induced hippocampal inflammatory injury.^63^ Whole N6‐methyladenosine (m6A) RNA methylation in the hippocampus of aged POCD mice constructed using sevoflurane, affecting gene expression.^64^ Low‐dose sevoflurane pretreatment of rats alleviates CPB‐impaired cognitive function and induces apoptosis in hippocampal cells, and the phosphatidylinositol 3 kinase (PI3K)/protein kinase B (AKT) signaling pathway is inactivated in hippocampal tissues of CPB‐POCD rats by low‐dose sevoflurane treatment.^10^ In brain tissue 24 h after isoflurane anesthesia, the hippocampal western‐blot method detected increased expression of many inflammatory factors NLRP3, IL‐1β, and IL‐18.^29^ RNA‐seq in the hippocampus of young mice anesthetized by repeated inhalation has also been found to be abundant in biological processes in brain development, learning, and memory.^22^

Dexmedetomidine (DEX) attenuates hippocampal neuronal apoptosis, neuroinflammation, DNA damage, and cognitive impairment in older mice and has potential neuroprotective effects.^65^ DEX has been shown to reduce inflammation around the heart and hippocampus after CPB surgery in rats.^66^ DEX exerts neuroprotective effects by inhibiting inflammation, apoptosis, and microglial activation in the hippocampus's prefrontal integument and dentate gyrus (DG) region.^67^ As an adjunct to anesthesia, DEX increases levels of biomarkers matrix metalloproteinase‐12 (MMP‐12) and myelin basic protein (MBP), all of which indicate that DEX is beneficial.^68^

Increased intraoperative intubation time is a major risk factor for acute cognitive decline.^52^ Prolonged anesthesia triggers neuroinflammation and complement‐mediated synaptic phagocytosis of microglia, which pathologically leads to synaptic elimination in the SIN.^16^ Hyperoxia CPB, direct damage caused by reactive oxygen species in CABG patients, and normoxia CPB reduce myocardial oxidative stress (OS),^69^ due to a reduction in signaling pathways of OS, inflammation, and apoptosis by normoxia treatment.^70^ Hypothermia has a neuroprotective effect on POCD.^71^ Moderate hypothermia is better suited to current clinical practice as it reduces CPB time and complications compared to deep hypothermia.^72^, ^73^ Mild anesthesia promotes better neurocognitive recovery after surgery.^74^ On the contrary, prolonged anesthesia activates the NF‐κB inflammatory pathway, triggering neuroinflammation, suppression of neuronal excitability, cognitive dysfunction, and anxiety‐like behavior.^16^

### 4.3 Circulatory perfusion

There is controversy about whether intraoperative hypotension is associated with postoperative cognitive impairment and whether intraoperative hypotension prolongs ICU length of stay but does not increase mortality, hospital stay, and duration of mechanical ventilation (MV). Moller et al. performed the neuropsychological examination in 1218 patients older than 60 years before and 1 week and 3 months after major noncardiac surgery and also showed that hypotension is not an important risk factor at any time.^50^ There was no significant correlation between intraoperative hypotension and the incidence of POD or POCD.^75^

However, hypotension may cause insufficient perfusion, insufficient perfusion is closely related to the presence of POCD. Microcirculatory perfusion impairment is commonly seen in patients undergoing CPB cardiac surgery. By examining the impairment of cerebral flow autoregulation (CA) during CPB cardiac surgery, the duration of the single longest CA injury event was found to correlate with the development of POCD. The duration of the single longest CA injury event was a risk factor associated with POCD.^3^ CPB during cardiac surgery mainly impairs microcirculatory flow without affecting total microvascular volume.^76^ Cerebrovascular blood flow is related to cerebral perfusion and directly affects postoperative recovery, but there is no effective way of cerebral perfusion.^77^

## 5 POSTOPERATIVE RISK FACTORS FOR POCD

In elderly patients undergoing CABG or valve transplantation, patients who experience POD have lower cognitive function on the day of surgery, at discharge, and at 1, 6, and 12 months after surgery. Self‐assessed depression is more likely to occur 1 week after surgery in patients with POCD. Patients with insulin resistance (IR) are more likely to develop POCD more than 1 week after surgery. In other types of surgery, such as abdominal surgery, postoperative infections, and pulmonary complications are more likely to occur in patients with POCD. After total hip arthroplasty, decreased hematocrit levels, and POCD is more likely to occur within 24 h after surgery. In elderly patients aged 60 or above who undergo total hip arthroplasty and receive patient‐controlled analgesia after surgery, the group receiving hydrocodone shows significantly higher scores on the Mini‐Mental State Examination (MMSE) compared to the group receiving sufentanil. POCD occurs on postoperative Days 1, 3, 5, and 7.^78^

During cardiac surgery, CPB impairs sublingual microcirculatory perfusion. Functional capillary density (FCD), perfused vessel density (PVD), and proportion of perfused vessels (PPV) are reduced. Microcirculatory perfusion disturbances persist for at least 24 h after surgery, and long‐term impairment of microcirculatory perfusion occurs postoperatively.^76^ Xie et al. found that an increased length of stay (LOS) in the ICU and the presence of arrhythmias, including atrial fibrillation (AF), are also risk factors for postoperative cognitive decline (Table 3).^52^

**Table 3:** The risk factors for postoperative cognitive dysfunction (POCD).

Preoperative risk factors	Intraoperative risk factors	Postoperative risk factors
Age Education level DM ASA score Others Preoperative depression, Physical weakness	Operation mode Anesthetics Anesthesia method, duration, depth of anesthesia Intraoperative hypotension Cerebral perfusion	Postoperative complications and infection IR Depression Hematocrit levels Analgesic use Microcirculatory perfusion Increased days in the ICU

Abbreviations: ASA, American Society of Anesthesiology; DM, diabetes mellitus; IR, Insulin resistance.

## 6 MONITORING INDEX

### 6.1 Cognitive assessment tools

Neuropsychological assessment is the gold standard for POCD diagnosis, and simplified tests are more commonly used in trials and clinical practice, but modified telephone interviews of cognitive status and Montreal cognitive assessments are less prevalent than outcomes following neuropsychological assessment.^79^ Cognitive measures reflect vulnerability to cerebral under‐perfusion and provide objective and timely preoperative health indicators to inform postoperative interventions to optimize the prognosis of surgical patients.^80^, ^81^ The high prevalence of POCD in patients undergoing elective vascular surgery and poorer cognition are closely associated with POD, and screening for cognitive impairment is necessary as a routine preoperative monitoring program.^82^

### 6.2 Regional cerebral oxygen saturation

Intraoperative cerebral oxygen saturation monitoring is a noninvasive method, and a comprehensive assessment of rSO_2_ guides interventions for cognitive outcomes after cardiac surgery. Determining whether the intervention guided by intraoperative monitoring of rSO_2_ can reduce the risk of POCD is controversial. However, evidence from a study of cardiac surgery patients suggests that reduced rSO_2_ is associated with an increased risk of POD, that preserving blood flow to the brain is associated with fewer adverse outcomes after direct cardiac surgery, and that rSO_2_ monitoring‐guided interventions reduce the risk of POD and POCD and shorten the length of ICU stay in surgical patients.^43^ A retrospective analysis of a large sample compared that rSO_2_ monitoring during surgery was a major predictor of all complication outcomes. Patient rSO_2_ status was associated with lower stroke, renal failure requiring dialysis, and mortality.^83^ neurological complications were more pronounced in CABG patients, with a 20% and greater reduction in rSO_2_ during CPB.^84^ Monitoring the range of intraoperative rSO_2_ decreases can predict POCD, and rSO_2_ is important as a routine monitoring item in elderly patients undergoing cardiac surgery.^85^

### 6.3 MedicalImaging

A retrospective study found a higher incidence of POCD in patients undergoing elective cardiac surgery with cerebral infarction, carotid stenosis, and intracranial artery stenosis detected by preoperative magnetic resonance imaging (MRI) and MR angiography as risk factors for POCD in these middle‐aged patients.^86^

### 6.4 Other potential biomarkers

Perioperative neuropathological processes are complex and diverse, and it is difficult to reveal the pathogenesis of diseases by a single research method. In recent years, new research methods such as microarrays (gene chips) have been a fast, efficient, and comprehensive method widely used to diagnose diseases, especially in hereditary diseases, chromosomal abnormalities, mutations, infectious diseases, and disease‐related biomarkers. The use of microarray‐based methods facilitates the analysis and study of POCD.^87^ Suo et al. found in RNA‐seq analysis of the hippocampus of sevoflurane‐anesthetized aged mice that differentially expressed genes (DEGs) were increasingly present over time as the surgery began to end early and late.^4^ Yazit et al. through screening 17 high‐risk coronary artery bypass graft patients aged 40–75 at the genetic level, a large number of differentially expressed genes were identified.^88^ Screening potential genes and transcription factors for POCD by bioinformatics analysis, most of the potential target genes are involved in the regulation of transcription factor (TF) TFs, which may have an important impact on the development of POCD.^89^

Some evidence suggests that noncoding RNA (ncRNA) may play an important role in POCD. microRNA, lncRNA, and circular RNA (circRNA) can regulate POCD‐related processes, and ncRNA may be a biomarker for POCD diagnosis, treatment, and prognosis.^12^ Microglia activation during surgery and the subsequent elevated release of inflammatory cytokines in the brain predispose individuals to POCD. mir‐124 is one of the most abundant micro RNAs regulating microglia function in the brain.^90^ miR‐34a, miR‐15a, and miR‐320a may be jointly involved in developing PND after cardiac surgery.^91^ miR‐124‐3p overexpression protects against PND, alleviating inflammation, reducing apoptosis, and repairing cognitive function.^92^ Elevated serum lncRNA‐MYL2‐2 and decreased miR‐124‐3p are associated with decreased cognitive function in patients.^44^ Sevoflurane‐induced POCD‐associated exosome delivery mir‐584‐5p may regulate (microglia) HMC3 cell growth by targeting BDNF.^93^ Perinatal fluorosis affects learning memory capacity also due to enhanced miR‐124 and miR‐132.^94^ Plasma miRNA‐221‐3p can be used as a biomarker of major depression or mood, and miRNA‐221‐3p may also be a valid predictor of postsurgical POCD.^95^ A large number of circRNAs with significant differential expression in the transcriptome of the hippocampus of POCD mice, which regulate cognitive function, maybe a new diagnostic biomarker and therapeutic target for POCD.^96^

The role of sirtuin 1 (SIRT1) in neuroinflammatory initiation and cognitive deficits in aged rats after anesthesia and surgery. SIRT1 plays a key role in several different neurological disorders through its involvement in microglia activation. Age‐related SIRT1 regulates cardiac metabolism elevates serum NLRP3 protein levels during ischemic and reperfusion (I/R) stress, and increases the risk of PND 7 days after surgery. SIRT1 attenuates NLRP3 inflammasome‐dependent inflammation and cellular scorching during myocardial I/R through metabolic regulation, and SIRT1 overexpression is cardioprotective.^97^, ^98^, ^99^ Hippocampal expression of SIRT1 decreases with age, and the trend of decreasing SIRT1 expression is further worsened after surgical anesthesia in aged rats.^100^ Cognitive performance was assessed using the Y‐maze and Morris water maze tasks (MWM), and SIRT1 expression levels were downregulated in POCD.^101^ Microglia‐derived neuroinflammation induced by SIRT1 inhibition may exacerbate tau acetylation in cultured neurons in vitro.^102^ SIRT1 may also play a role in stellate ganglion block (SGB), an effective intervention for POCD that enhances SIRT1 expression and decreases NF‐κB activity in the hippocampus and white matter, and it increases the levels of inflammatory factors in serum and white matter, mainly at the level of anti‐inflammatory factors.^103^ Obese mice exacerbate postoperative hippocampal‐dependent cognitive dysfunction via the SIRT1 pathway.^104^ Repeated administration of isoproterenol to neonates impairs learning and memory abilities, also due to reduced SIRT1 expression, inhibition of synaptic plasticity, and reduced excitability of glutamatergic neurons in the hippocampus.^105^ Hyperbaric oxygen (HBO) preconditioning increased SIRT1 and improved memory dysfunction.^106^ Overexpression of the important autophagy‐regulated AMP‐activated protein kinase α1 (AMPKα1) upregulates SIRT1 expression in the hippocampus of POCD rats, which improves POCD.^107^ These suggest that SIRT1 has an important neuroprotective role in the pathogenesis of POCD and is a potential molecular target for POCD treatment.

Glial fibrillary acidic protein (GFAP) is one of the sensitive biomarkers of brain injury, and elevated GFAP serum is associated with cognitive impairment. Widya et al. found significantly higher postoperative GFAP levels in POCD patients than in non‐POCD patients through a study of 56 subjects who underwent elective CABG pump surgery.^108^ Soluble trigger receptors expressed in the CSF of patients with early cognitive dysfunction after thoracoabdominal aortic coarctation have increased myeloid 2 (sTREM2) concentrations, and sTREM2 is associated with early cognitive dysfunction after surgery in patients undergoing cardiac surgery.^109^ These findings facilitate further understanding of the pathogenesis of POCD and may be a new biomarker for diagnosing and monitoring POCD as a new therapeutic target for POCD (Table 4).

**Table 4:** Main monitoring indicators and potential biomarkers of POCD.

Type of monitoring indicators	Diagnostic methods	Advantage	Disadvantage
Cognitive assessment tools	Modified telephone interviews of cognitive status Montreal cognitive assessments Neuropsychological assessment	Simple and easy Simple and easy Gold standard	Less prevalent than outcomes following neuropsychological assessment Less prevalent than outcomes following neuropsychological assessment Complicated and time‐consuming
rSO_2_	rScO_2_	A noninvasive method	Increase medical expenses
MedicalImaging	MRI and MR angiography	Predicting the risk factors for POCD in middle‐aged patients in advance	Increase medical expenses
Other potential biomarkers	mir‐124, SIRT1, GFAP	May become a new therapeutic target for POCD	Additional testing is required

Abbreviations: GFAP, glial fibrillary acidic protein; POCD, postoperative cognitive dysfunction; rSO_2_, regional cerebral oxygen saturation; rScO_2_, regional cerebral oxygen saturation; SIRT1, sirtuin 1.

## 7 TYPES OF THERAPY

Currently, the pathogenesis of POCD after heart surgery with CPB is not well understood, and the available treatment options are limited. Commonly used pharmacological treatments include DEX, which has anti‐inflammatory and antioxidant properties. DEX has been shown to significantly reduce apoptosis in the hippocampal cells of elderly mice induced by surgery, thereby exerting neuroprotective effects against surgery‐induced cognitive impairment.^110^ Nonsteroidal anti‐inflammatory drugs such as acetaminophen (APAP) have neuroprotective effects through their antioxidant and anti‐inflammatory properties, as well as their ability to inhibit the MPT pores and subsequent apoptotic pathways.^111^ Commonly used perioperative antibiotics, such as cefazolin, can reduce intestinal dysbiosis caused by anesthesia and surgery, prevent disruption of the BBB, and improve POCD.^112^ Opioids such as ketamine can attenuate POCD after cardiac surgery by inhibiting the stimulator of interferon genes (STING) and TANK‐binding kinase 1 (TBK1) signaling pathway.^113^ Local anesthetics such as lidocaine can attenuate the occurrence of POCD induced by inhalation anesthesia with sevoflurane by reducing mitochondrial damage and altering the ratio of the apoptosis‐promoting protein (Bax) to the apoptosis‐inhibiting protein (Bcl‐2).^114^ Starting from relevant risk factors, early intervention, prevention of irreversible damage, and perioperative treatment and prevention are effective strategies to avoid the occurrence of POCD.

## 8 CONCLUSION

**Figure 2:**
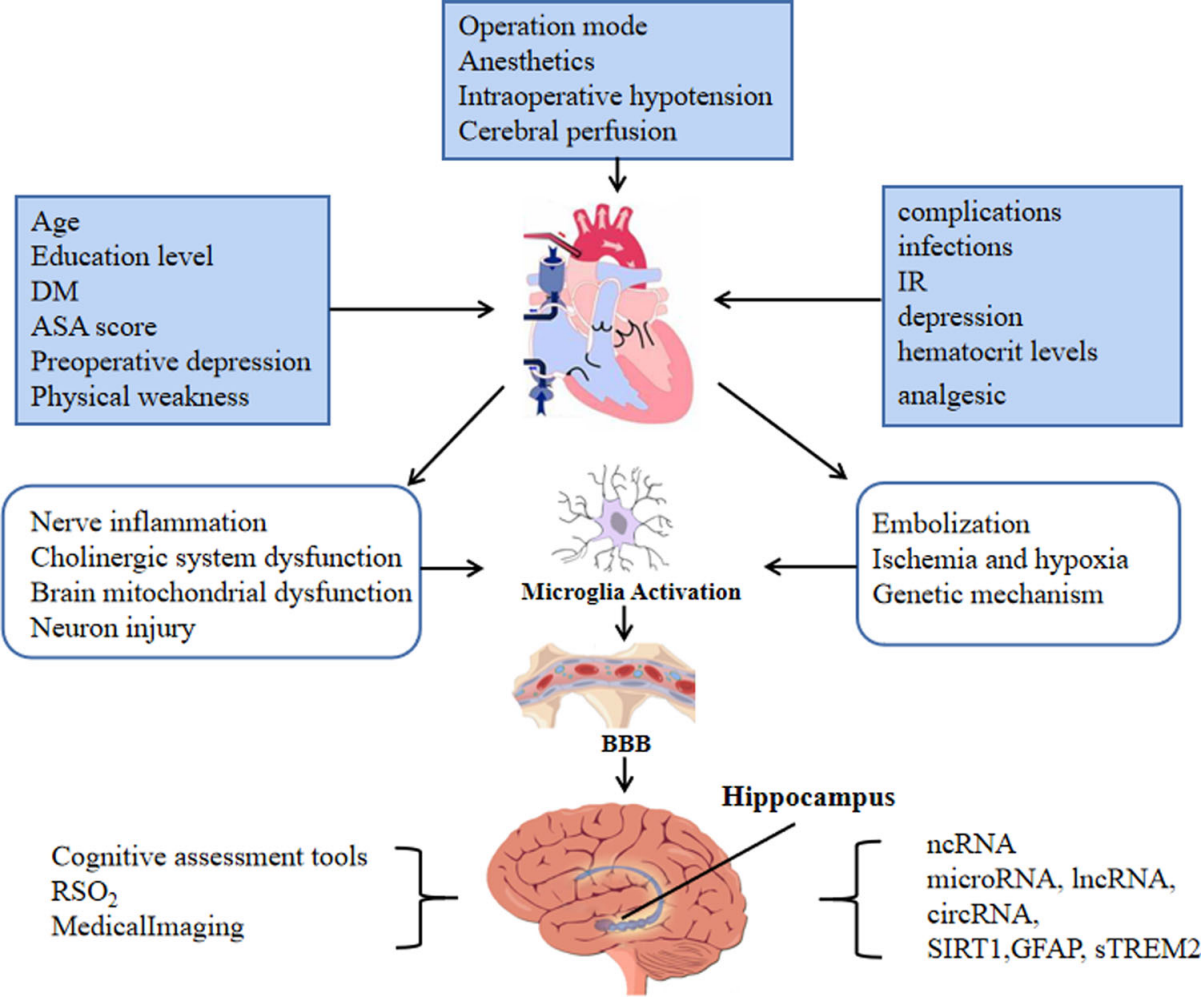
Research progress of postoperative cognitive dysfunction in cardiac surgery under cardiopulmonary bypass (CPB). CPB is used in cardiothoracic surgery and leads to pathophysiological changes in the body, causing multiorgan and multi‐tissue damage and frequently leading to central nervous system complications. ASA, American Society of Anesthesiology; DM, diabetes mellitus; GFAP, Glial fibrillary acidic protein; IR, insulin resistance; ncRNA, noncoding RNA; RSO_2_, regional cerebral oxygen saturation; SIRT1, sirtuin 1; sTREM2, thoracoabdominal aortic coarctation have increased myeloid 2. [Color figure can be viewed at wileyonlinelibrary.com]

In summary, with the development of science and technology, there has been substantial progress in surgical and anesthesia techniques, but the incidence of POCD in patients undergoing cardiac surgery with extracorporeal circulation is still higher than that of other surgical modalities. POCD after heart surgery with CPB is the result of various factors, with intraoperative direct cardiac surgery and elderly patients being important risk factors for POCD. At present, there are limited treatment options for POCD. Early prevention, shortening anesthesia time, individualized selection of anesthetic drugs, strengthening intraoperative monitoring, and the use of anti‐inflammatory drugs are recommended. Reducing the incidence of POCD after cardiac surgery is beneficial to the development of medical care and is one of the effective ways to solve the conflicts between patients, their families, and hospitals and reduce the burden on society. Neuropsychological evaluation remains the gold standard for stable and accurate diagnostic assessment. In the future, we should make more use of microarrays (gene chips) and RNA‐seq analysis, combined with bioinformatics analysis of potential targets, to explore the pathogenesis of POCD at the gene level, and to find effective methods for preventing and treating POCD (Figure 2).

## AUTHOR CONTRIBUTIONS

Kun Zheng and Hong Zhang contributed to the central idea. Ji‐Yang Xu and Yi‐Ming Zhuang completed the literature search, collation, and paper writing. Hong Zhang and Yi‐Ming Zhuang revised the manuscript.

## CONFLICT OF INTEREST STATEMENT

The authors declare no conflict of interest.

## ETHICS STATEMENT

Not Applicable.

## Data Availability

Data supporting this study's findings are available from the corresponding author upon reasonable request.
